# Wild Mammals in the Economy of Wrocław (Poland) as an Example of a Medieval and Modern Era City in the Light of Interdisciplinary Research

**DOI:** 10.3390/ani11092562

**Published:** 2021-08-31

**Authors:** Marta Pietruszka, Jerzy Piekalski

**Affiliations:** Institute of Archaeology, University of Wroclaw, Szewska 48, 50-139 Wrocław, Poland; jerzy.piekalski@uwr.edu.pl

**Keywords:** wild mammals, archaeozoology, economy, historical archaeology, hunting

## Abstract

**Simple Summary:**

The animals that held the greatest importance in the economy of a medieval and early modern city were domesticated species, such as cattle, pigs, goats and sheep. These animals were used as sources of meat, bone, horn and leather in crafting items for daily use; however, skeletal remains belonging to wild animals are also encountered during archaeological research. The purpose of this article was to determine the role of wild animals in the economy of a historical city on the basis of research conducted in Wrocław. The base material consists of bone remains belonging to various species, as well as items manufactured from the leather, horn and bones of wild animals. The collected information was compared with the current state of historical research. It turned out that the analysis confirmed the fact that wild animals played a small but constant role in the economy of medieval and early modern Wrocław from the 11th to the 17th century. The rare use of such materials might indicate occasional breaching of hunting laws and limitations functioning until the 15th c., the characteristics of the local environment with its low amount of wild game and the elite characteristics of wild animal meat and products.

**Abstract:**

The purpose of this article was to determine the role of wild animals in the economy of a historical city on the basis of archaeological and cultural layers of medieval and early modern Wrocław from the 11th to the 17th century. Archaeozoological analyses were applied, mainly encompassing the percentage share of particular animal species and the research of material culture, i.e., items manufactured from bones, antlers and hides of wild animals. The collected data were compared with written sources. As a result of the following analysis, a low but stable frequency of bone remains in urban layers and is evidence for occasional breaching of the medieval hunting laws by burghers, possibly driven by the opportunity to sell meat and other wild animal products on the markets. Moreover, the relatively low amounts of items made from bones, antlers and wild animal leather may indicate low availability or seasonality (shed antler) of the materials, which might have indirectly raised the product price. Additionally, the area around Wrocław did not feature large forest complexes, which are habitats of wild game, thus explaining the low frequency of wild animal remains in the archaeozoological material.

## 1. Introduction

Archaeozoology is an important branch of the study of the past. In the case of medieval and modern cities, it allows for the verification of information about animals contained in written sources, which inform us about the use of animals in the urban economy, their social role and the symbolic meaning of particular species. Sometimes, the written sources are insufficient, and archaeozoological research can fills gaps in the current knowledge. In addition, written sources may present an incomplete story, and the information they contain may be biased. A good example of a city with a rich history but incomplete written sources with respect to the discussed issue is Wrocław.

The consumption of meat by the inhabitants of cities located in the modern Polish territory, including Wrocław, is an issue frequently discussed in the literature [1,2,3,4,5,6,7,8,9,10,11]. Animals that hold the most economic importance in medieval cities are domesticated animals, i.e., cattle, pigs, goats and sheep. Their remains, in the form of skeletal material acquired from research, have varied in amount depending on the historical period and local economic conditions. Wild animal remains usually constitute only a small percentage of the whole. Moreover, according to written sources, the consumption of wild animals was marginal, mainly due to legal regulations that limited access to the wild game to only the local ruler and his closest retinue [2,4,12,13,14]. In spite of this, archaeological research across the ages shows that the use of wild animals has its permanent place in the economy.

The purpose of this article is to present the role of wild animals in the economy of a historical city. The main source base comprises the archaeological material acquired from the medieval and early modern cultural layers of Wrocław, the capital of Silesia, a historical area located in modern-day southwestern Poland; however, completing such a task requires using methods and sources from several other disciplines, mainly archaeozoology, studies of material culture and the analysis of written sources. Archaeozoological research is particularly important here, directed at recognising the animal species that were used and reconstructing the structure of age, sex and anatomical layout of skeletal remains [5,15]. Written texts, such as hunting manuals and poems, are also a priceless source of information about the importance of wild animals in the economy and culture. Particularly important for this article is information gathered by Agnieszka Samsonowicz in her work Hunting in Poland in the Times of the Piast and Jagiellonian Dynasties, focusing on the social, cultural and economic phenomena related to hunting [16].

An important issue is the way in which pieces of wild animal bodies were used in manufacturing items of daily use, such as items made of leather, bone and horn, which are found at the archaeological sites and represent evidence of human activity [5]. Such items are quite frequently researched in archaeology [17,18,19]: and the references therein. Papers in that field often focus on the manufacturing technology, used materials, or, more broadly, the functioning of workshops that processed hides, leather and horn.

### Brief Historical Outline of Wrocław

**Figure 1:**
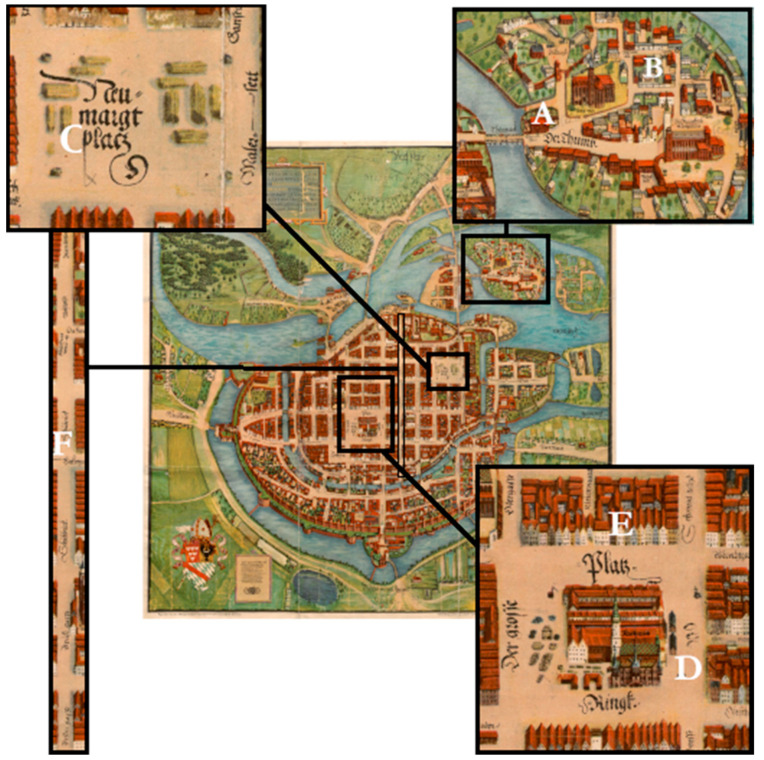
Plan of the late medieval Wrocław and location of the discussed sites (Contrafactur der Stadt Breslau 1562, Barthel Weiner) oai: www.bibliotekacyfrowa.pl:39530 (accessed on 5 August 2021). Description: M. Pietruszka. A: Katedralna St., Ostrów Tumski (Cathedral Island); B: św. Idziego St., Ostrów Tumski (Cathedral Island); C: Nowy Targ Square; D: Market Square; E: Market Square 50-Igielna St. 18; F: Szewska St.

Wrocław was one of the most significant urban centres in Silesia. Presenting its brief history will allow for identifying the differences in the city’s sociotopography and the pace of its development from the early Middle Ages until the modern era. The foundation of Wrocław can be dated to the mid-10th century. At that time, an island located on the Oder and later named Ostrów Tumski (Cathedral Island), was founded as a fortified settlement. In 1000, the settlement became the seat of a bishopric. The settlement then became a seat of secular and church power, both heavily influencing the way the city developed [20,21,22]. Wrocław grew rapidly, mainly in the 12th and 13th c., which was directly linked to the demographic and economic growth and, as a consequence, the need to relocate the population from the overcrowded area of Ostrów Tumski. The commune settled the large area located on the western bank of the Oder (Figure 1) [23]. Two German law location charters (the first one in 1242 and the second one in 1261) brought a wave of new settlers (mainly German) and further confirmed the role of Wrocław as the dominant economic and political centre in Silesia [24]. Ostrów Tumski was inhabited by clergy, nobles and members of the ducal administration until the end of the 13th century. This changed in the 14th century, when the island became the sole property of the church [24,25]. The last of the local dukes, Henry VI, died in 1335, and the city became the property of the kings of Bohemia and one of the most important cities in the kingdom after Prague. The city had quite a substantial autonomy, and its economy boomed [26,27]. In 1526, Ferdinand Habsburg was elected the new king following the death of the Bohemian–Hungarian king Louis in the Battle of Mohacs. This was supposed to strengthen the state and guarantee potential support from the Holy Roman Empire and Spain in case of Turkish aggression. The election of Ferdinand was the beginning of Habsburg rule in Wrocław, which lasted for over two centuries [28].

## 2. Materials and Methods

The archaeozoological and archaeological material used in this analysis was acquired during archaeological research conducted over 20 years in various parts of Wrocław, namely the fortified settlement in Ostrów Tumski and in the urbanised area on the left bank of the river Oder (Table 1, Figure 1). The results were collected from the published research of animal remains, mostly as parts of broader archaeological works at given sites. The collected data were collected and brought together from individual studies published in the literature and presented as graphs and tables, focusing mainly on the percentage amount of particular animal species. As a result of visual comparative analysis, the total number of fragments (TNF) and the number of identified specimens (NISP), species composition and anatomical layout were identified [1,29,30,31,32].

The archaeozoological research included an osteometric analysis conducted in accordance with the von den Driesch methodology [33]. Moreover, age profiles were drawn using the methods of Zietschmann, Kröling [34], König, Liebich [35] and Reitz, Wing [36]. Additionally, the anatomical layout of post-consumption waste was prepared for the domesticated animals in accordance with Reitz, Wing [36]. The methods used along with their corresponding study are listed in Table 2. Unfortunately, the majority of the broader, abovementioned analyses were conducted on the remains of domesticated animals, which prevents any deeper conclusions regarding wild animals. Such a situation has resulted from the high level of fragmentation of skeletal remains, and the sample sizes were insufficient for statistical analyses; therefore, this article uses the analyses of relative amounts of remains and their percentage share in the identified assemblage of animal bones. The percentage was ordered chronologically from the 11th to the 17th c., which allowed us to trace the changes in the appearance of particular species of wild animals. Moreover, the social differences between Ostrów Tumski (an elite area) and the settlement on the left riverbank (occupied by traders and artisans) are highlighted.

Studies on material culture which focus on finished products or their fragments from leather, bone and antlers of wild animals were also taken into consideration (Table 3). They are the results of published research that was a part of broader, interdisciplinary projects. Expertise species identification of leather finds was conducted using a stereoscopic microscope. The grain pattern morphological analysis of the leather surface allowed us to identify the species of animal whose skin was used to manufacture the item. In the case of raw and severely damaged hides, such analysis is impossible [37,38]. The items made from bone and antler were, similarly to their leather counterparts, published in the literature and identified via a visual comparative method [39,40,41].

**Table 2:** List of methodologies used in the archaeozoological research according to the authors of the publications. Description: M. Pietruszka.

Location	Literature	Methodology
Nowy Targ Square	Chrószcz, Janeczek Pasicka 2018 [1]	Von den Driesch 1976 [33]—osteometric research
		Zietschmann, Krölling 1955 [34]; König, Liebich 2008 [35]; Reitz, Wing 2001 [36]—age profile
		Reitz, Wing 2001 [36]—anatomical distribution of post-consumer remains of domestic animals
św. Idziego Street, Ostrów Tumski (Cathedral Island)	Chrószcz, Janeczek, Poradowski, Sudoł 2015 [30]	Von den Driesch 1976 [33]—osteometric research
		Zietschmann, Krölling 1955 [34]; König, Liebich 2008 [35]; Reitz, Wing 2001 [36]—age profile
		Reitz, Wing 2001 [36]—anatomical distribution of post-consumer remains of domestic animals
4 Katedralna Street, Ostrów Tumski (Cathedral Island)	Chrószcz, Janeczek 2012 [29]	Von den Driesch 1976 [33]—osteometric research
		Zietschmann, Krölling 1955 [35]; König, Liebich 2008 [35]; Reitz, Wing 200 [36]—age profile
		Marciniak 2003 [42], Lasota-Moskalewska 2008 [43]—analysis of traces of human activity
Rynek (Market Square)—18 Igielna Street	Wiszniowska, Stefaniak, Socha 2002 [32]	Habermehl 1975 [44]; Zietschmann, Kröling 1955 [34]—age profile
		Von den Driesch 1976 [33]—osteometric research
		Uerpmann 1973 [45]—method of counting the analysed remains
Rynek (Market Square)	Wiszniowska, Stefaniak, Socha 2001 [31]	Habermehl 1975 [44]; Zietschmann, Kröling 1955 [34]—age profile
		Von den Driesch 1976 [33]—osteometric research
		Uerpmann 1973 [45]—method of counting the analysed remains

Apart from the analyses mentioned above, written sources in the literature were also taken into consideration, mainly those covering the issue of hunting limitations and privileges in the Middle Ages [16,47,48] as well as general practices and traditions connected with hunting [16,49].

## 3. Results

**Figure 2:**
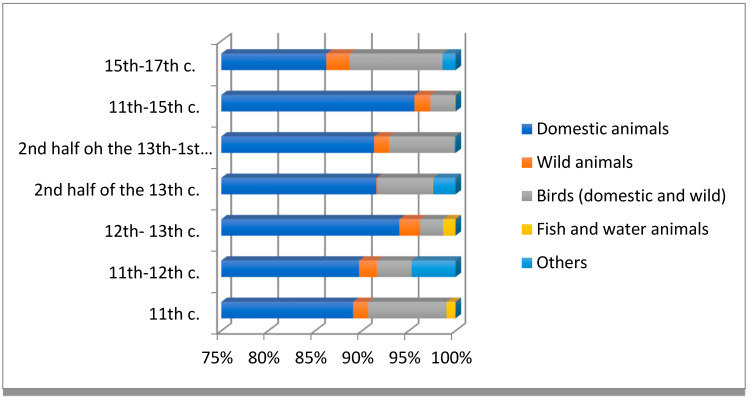
The bones of individual groups of animals present in medieval and modern Wrocław according to results of the discussed archaeozoological research [1,29,30,31,32]. Description: M. Pietruszka.

**Figure 3:**
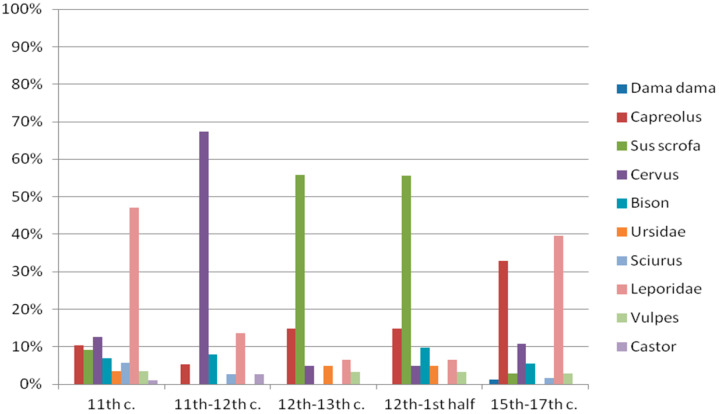
Species share of wild animals in Ostrów Tumski (Cathedral Island) over the centuries [29,30] (species names in Latin). Description: M. Pietruszka.

Animal remains acquired from 4 Katedralna Street at Ostrów Tumski were split into two chronological phases: from the 11th to 12th c. and from the 15th to 17th c. (Figure 2 and Figure 3) Due to the relatively low fragmentation of the remains, circa (ca.) 6% of the material was identified (9015 fragments) [29]. In the case of the first phase, 1.9% of the identified remains came from wild animals. The bones belonged to the following species: roe deer, red deer, wisent, squirrel, hare and beaver. Most of the bone fragments were pieces of ribs, shoulder blades and humerus, with a few fragments of other limb bones. In phase II, wild animal bones constituted 2.5% of the assemblage, with such species as roe deer, red deer, fallow deer, wisent, squirrel, hare, fox and boar. Most of the bones were fragments of vertebrae, shoulder blades, pelvis and tibia. Additionally, pieces of skulls and limb bones were found [29].

Research was also conducted at a site located at 4–6 Św. Idziego Street at Ostrów Tumski (dig site no. IIIF). The material was split into two phases, the first one dated 10th–11th c; however, the layers dated for the 10th c. contained remains of only domesticated animals. The second phase is dated from the 12th to the first half of the 13th c.—the condition of the remains allowed for ca. 54% (8371 fragments) to be identified. In phase I, wild mammals constituted 1.5% of the identified material, mostly represented by hare bones, followed by red deer, roe deer, boar, wisent, squirrel, fox, bear and beaver. In the case of phase II, there was a slight increase in the material from wild animals: 2.2%. Bones of boar, roe deer, wisent, hare, bear, red deer and fox were found, with boar bones constituting over half of the assemblage (Figure 2 and Figure 3) [30].

**Figure 4:**
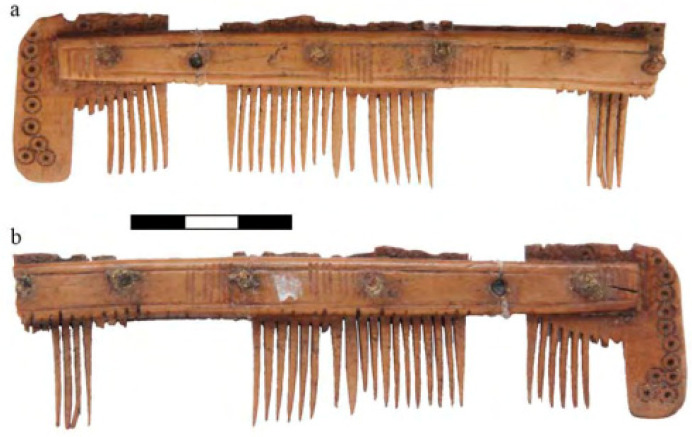
Wrocław—Ostrów Tumski (Cathedral Island), No. 4–6 św. Idziego Street, trench IIIF. Deer antler comb, layer E2, catalogue No. 548a/01. Photographs of both sides of the artefact (**a**,**b**). Photo by K. Jaworski [41].

Items made from bones and antlers found at Św. Idziego Street at Ostrów Tumski came from layers dated for the 11th c. Apart from some identified items and fragments of raw material, some artifacts were identified. One of the most interesting finds was a comb (Figure 4) made from deer antler, whose presence is proof of contact with the settlements of the Western Pomerania. Such provenance is indicated by the form and ornamentation of the comb. The comb, despite its fragmentary preservation, was typologically analysed and identified as a representative of group IB, type VII, variation 6, subvariation c, in the classification by Cnotliwy (1973), i.e., a single-sided comb with narrow, arched fittings and toothed plates with wavy top edges, decorated with a row of holes [41]. Additionally, other items were identified, such as a tourniquet made of deer antler, conical fittings that may have been used to reinforce knife handles, a small arrowhead made of deer antler and bone (bear or horse bone) and antler (red or roe deer) spikes [41]. Św. Idziego Street also yielded numerous leather fragments. Their condition, however, prevents any precise identification. A piece of goat or deer leather, decorated with embroidery, comes from the third quarter of the 11th c. A leather scrap from a cut shoe vamp is dated for the turn of the 12th c. [37].

The site at 4 Katedralna Street yielded two fragments of early medieval deer antler with traces of processing [40] as well as a deer antler plug from the 16th c., bearing elongated carved lines beneath the top crudely carved groove [40].

The condition of the bone remains from the main Market Square (Rynek) in Wrocław, within the judicial square (dig site no. IX), is varied but mostly fragmented (1805 identified fragments). The material bears traces of processing and carving. Almost 46% of the remains were attributed to particular species predominantly belonging to domesticated animals [31]. In this context, some hare bones were identified, constituting only 0.5% of the whole material. Most of the remains were skull fragments, with individual pieces of shoulder blade, femur, heel bone, humerus, pelvis, metacarpal and metatarsal bones [31]. Additionally found in dig no. VII/7, in layers dated for the 2nd half of the 14th c., were numerous fragments of processed deer antler as well as ribs and shoulder blades. The presence of such remains suggests the workshop of a horn carver (Figure 2) [31].

In the site located at Rynek 50-Igielna 18 in the northern part of the main square, only about 49% (4369 fragments) of the material from the second half of the 13th c. was identified, mainly due to heavy fragmentation of the bones. The layers yielded six fragments of hare bones: mainly from the skull, as well as individual pieces of pelvis, tibia and heel bone (Figure 2) [32].

Nowy Targ was one of the three market squares of Wrocław. It is located in the eastern part of the city and was a trading and manufacturing hub. The archaeozoological material acquired from this site was far less fractured than the pieces from the main market square. Species were identified in the case of 72% of the material (5894 fragments) [1]. The remains that were wild animals constituted only 1.6% of the whole identified material. The identified species were boar, roe deer, red deer, hare, wisent, fox and squirrel [1]. Anatomically, the identified fragments included pieces of skulls, ribs, shoulder blades and, most commonly, pieces of limb bones [1], Figure 2.

The layers at Nowy Targ were dated from the 11th to the 18th c. Of the 1136 leather fragments, species were identified in 926 of cases. Leather from wild animals was used in the case of 87 fragments. The most popular material coming from wild animals was deerskin. Leather fragments constituted 7.66% of the whole analysed assemblage. Parts of gloves, shoes (e.g., bootlegs, gussets and heel counters), belts, pouches and sheaths were identified. Apart from deerskin, individual fragments of fox leather were found as well as a fragment of a knife sheath made from beaver tail leather [50]. Thanks to the precise dating of layers encompassing a broad chronological spectrum, it was possible to determine the share of wild animal materials in particular phases. The oldest find, a fragment of a deerskin glove, was dated for the 12th c. In the phase dated for the second half of the 12th and the beginning of the 13th c., wild animal material fragments constituted only 4.97% of all finds from that phase, represented mainly by shoe fragments and deerskin scraps. The finds from the 13th c. include fragments of shoes, sheaths, belts, leather scraps and production waste and constitute 10.39% of the assemblage. At the turn of the 13th c., the number of such materials decreased to 7.42% and is represented mainly by shoe fragments, scraps and waste as well as by fragments of a pouch, belt and pattens. The lowest frequency of wild animal leather (3.9%) is observed in the 14th c. and includes a strap, shoe fragments, production waste and a belt fragment. At the turn of the 14th c., the amount of deerskin significantly increased to 13.39%, represented by fragments of shoes, a belt, a sheath and numerous scraps. In the period dated for the 15th–17th c., wild animal leather constituted only 5.21% of all finds, represented by deerskin scraps, a piece of fox leather and the already mentioned sheath made from beaver tail ([38]: Annex XI.4).

Nowy Targ also yielded items made from deer antler that were related to entertainment—namely gaming pieces. Four circular pieces, decorated with carved circles and holes, were found in layers dated for the 13th/14th c. They were probably used in board games, such as backgammon or nine men’s morris [39].

Szewska Street yielded numerous leather fragments dated from the second half of the 13th c. to the first half of the 14th c., among which were those made from wild animal hides, which constituted 9.72% of the whole assemblage. Identified among them were fragments of footwear (vamps, bootlegs and heel counters), a sheath and three fragments of a mitten made from deer or elk skin [46].

## 4. Discussion

### 4.1. Hunting

Hunting was an important element of European culture in the Middle Ages [49]. It served as a kind of maturity exam for young men from the upper classes who honed their skills in using weapons, horse riding, survival and having a sense of direction [49,51]. Certainly, hunting had a more practical aspect as well, by providing meat, which was a rarity in certain times of the year, especially winter [49]. The animals also were hunted down in order to acquire fat, bones, teeth and hides. Antlers were used for manufacturing combs, buttons, blade grips, rosary beads, etc. Various parts of animals, such as deer hearts, were also used in medicine [16,49]. Hunting was, however, mostly a sport for the nobility rather than a simple chase for meat and fur. The cultural content significantly outweighed the economic aspect [16,49]. The final function of hunting was simply doing it for pleasure, although such entertainment was frowned upon by the Catholic Church, and the participation of the clergy in hunting was considered to be ungodly behaviour [48,49].

The ability to hunt was dependent on the accessibility to game, whose main habitat were woodlands, with some species also found in less wooded environment. Some information on the conditions for hunting in medieval Poland was provided in the 12th c. chronicle written by an anonymous author known as Gallus: “it is a densely wooded land, but [...] it is full [...] of bread and meat, fish and honey”. Poland is similarly described by the Bohemian chronicler Cosmas (1045–1125), who writes that the country is “rich with herds of beasts of burden and the woods are full of game” [52]; however, Wrocław and its nearest vicinity were not heavily wooded. Such landscape in this part of Poland is known as field-and-forest type. Small forests were present on the left side of the Oder, as suggested by forest-related place names (Gaj, Gajowice, Tarnogaj, Borek and Muchobór) [53]. In such areas, there was significantly less game, which resulted in legal regulation protecting it.

The issue of jura regalia—royal laws—and in this particular case, hunting laws, is discussed only occasionally in the literature, in which the very outdated opinion is often presented that since the tribal period, the right to hunt was limited only to the elders/nobility. Such customs were confirmed in written law through hunting privileges in later centuries, especially in the period of feudal fragmentation. Such a state supposedly lasted from the 10th to the 14th c. [16,47]; however, Agnieszka Samsonowicz [16,47] contests that opinion, claiming that the dynamic political, social, economic and ecological situation in the Piast monarchy directly influenced the application of hunting laws. Samsonowicz also points out that granting hunting privileges happened successively, in accordance with the needs of local rulers and, thus, it is impossible to generalise such processes. It should also be mentioned that the hunting regalia did not cover the whole country and only some of the grounds belonging to the duke, known in Polish as gaj or knieja or, in Latin, *forestes*, *gagium*, *gehagium regis* or *defensa*. Some of the local place names probably stem from such terms as Gajowice, Kniejniki, Łowicz and Leśnica [47]. The social importance of hunting is also visible in the presence of specialised professions related to hunting, such as falconers or marksmen. Their privileges grew with time, often leading to cases of power abuse. Agnieszka Samsonowicz points out that privilege documents only confirmed already-possessed rights and did not grant them, as was commonly thought [16,47]. During the 13th and 14th c., the local dukes, church officials and members of other elite groups competed for influence, which caused the hunting regale to disappear in the 15th c. [47]. This article focuses on the urban environment, and it should be noted that the participation of townsfolk in legal hunting was severely limited at least to the 15th c. The situation changed in favour of the burghers at the end of the Middle Ages [47].

Hunting was split into two categories: the great hunt or *venationes magnae*, and the small hunt, *venationes parvae*. The great hunt targeted large animals, categorised as *animalia magna* or *animalia superiora* (aurochs, wisents, bears, lynxes, elks and red deer), and the *parva* or *minuta* category contained smaller species (hares and roe deer). There were local variations in this classification depending on the composition of the local game. In the 13th and 14th c., with the local extinction of large animals, the value of *venationes parvae* increased [16,47]. Not only were hunting grounds and the types of game subject to limitations but also the most effective hunting methods: creating fenced areas with logs, using great deer nets and ceremonial hunting [16,47]. The local charters usually specified the prerogatives of local aldermen and sheriffs and the hunting privileges they held. Such privileges were usually used to satisfy personal needs, but the sources indicate that at least some of the hunted animals were sold for profit [47]. An example of this is the early 15th c. tax book from the Maltsters Quarter in Wrocław, which lists venison vendors, *wyltbreter* [54], who probably bought their stock from the local landowners. This premise is part of the described changes that begin with the disappearance of hunting laws. Unfortunately, little is known about the venison sellers. It can be assumed, on the basis of the tax book of 1403, that they did not fulfil a very substantial economic and social function. They lived in part of the southern section of the internal moat and belonged to lower-class taxpayers [54].

### 4.2. Archaeological Research

Our research shows that materials acquired from wild animals were rarely used in medieval Wrocław. Sample analyses of sources (food remains and crafting materials from hides, bones and antlers) from Ostrów Tumski and the urbanised area on the left bank of the Oder show general tendencies of how wild animals were utilised, which have remained low but constant over the centuries.

The presence of wild animals can be treated as a socioeconomic factor [55,56]. In the early Middle Ages, Ostrów Tumski was a seat of power and was inhabited by the upper classes, consuming larger amounts of wild animal products than the early urban area on the left bank of the Oder.

Wild mammals of the Middle Ages are mostly associated with a particular form of acquiring food—hunting, understood as a cultural phenomenon practised by the elite. Agnieszka Samsonowicz [47] stated that issuing hunting limits was mostly connected with the need to protect wild animals from being overhunted, especially in areas with a low population of game. The analysis of animal remains from various parts of Wrocław shows a low but stable presence of wild animals in the whole identified faunistic material across the ages (Figure 2). Such presence never exceeded 3%. A similar, stable situation can be observed in the case of other medieval and early modern cities in the region, i.e., Poznań [12] and Gdańsk [6]. The collected data suggest that the hunting laws were occasionally breached by the townsfolk.

By analysing the frequency of large animals (deer, bear and wisent) and smaller mammals (hare and fox), we were able to outline the features of the biotope that surrounded the archaeological site [30]. Some species inhabit only certain habitats; others easily adapt to environmental changes. According to Aleksander Chrószcz, Maciej Janeczek and others [57], heavily wooded areas were gradually replaced by arable land since the 12th c. Despite the fact that the composition of species represented in Ostrów Tumski shows some fluctuation over time, the presented data (Figure 3, Table 4) do not reveal any sudden changes, confirming the historians’ opinion about the mixed woodland–farmland character of the areas around Wrocław [53]. The presence of easily adapting species such as boars, hares and roe deer indicates the existence of fields, whereas red deer and wisent suggest the presence of woodlands. In the layers from the early modern era, we can observe a slight decrease in the amount of wild game, suggesting intensified development of settlements around Wrocław and growth in the farming economy [16]; however, it is worth noting that such conclusions should be drawn carefully due to the low percentage of wild animals in the identified material [29].

In the case of the site at św. Idziego Street (Cathedral Island), it was possible to anatomically analyse the wild animal species (Table 5) [29]. The anatomical analysis provides information on butchering practices and consumption preferences [55]. Due to the low number of remains, these data should be handled with great care [5]. Looking at the data, some trends can be seen for some species. The anatomical distribution of the hare suggests the use of the entire animal carcass, due to the presence of both the bones of the pectoral and pelvic limbs and individual bones of the head. A relatively higher number of deer head bones may indicate the acquisition of antlers. We also see a higher proportion of the pectoral limb of the roe deer, which may indicate a preference for this part of the carcass. The large proportion of the boar head bones indicates the consumption of this batch of carcass. More precise conclusions are unfortunately not possible here.

The urbanised part of Wrocław located on the left bank featured fewer remains of wild animals. Only hare bones have been found in the market square. They were represented by various anatomical parts. It can be assumed that the whole carcass was consumed [31,32]. In Nowy Targ Square, both the remains of larger and smaller animals such as deer, roe deer, wild boar, bison, hare, fox and beaver have been found [1], but due to the broad chronology of the researched material, it is impossible to make any specific conclusion. The fact that wild animal remains were found on market squares may confirm the local trade of such goods as meat, hides and bones. This is further confirmed by the entries in the tax register from the early 15th c., which lists venison vendors [54].

The use of hides, bones and antlers of wild animals is confirmed primarily by the artifacts found in various parts of the city. In the case of leatherware, deerskin was the most sought-after material, used for crafting such items as shoes, gloves, belts, pouches and knife scabbards. Deerskin is soft and durable, but often damaged due to wounds, scratches and parasite larvae [58].

Products made from wild animal hides constitute only a small percent of all finds. At Ostrów Tumski, they range from over 6% in the mid-10th c. to 3.7% in the first half of the 13th c. [37]. An analogous situation can be observed in the town of Puck, where 4.2% of all leather items were made from wild animal hides, encompassing similar categories of items [59]. At Nowy Targ, such items constituted over 9%. The largest number of such leather items appeared in the first half of the 13th c. and at the turn of the 14th c., including fragments of shoes and garments, pouches, scabbards and leather scraps and waste [50].

Items made from the bones and antlers of wild animals are quite rare and might have been treated as luxurious items [8]. Most of such items are made from various types of deer antlers, including both fragments of processed antlers and finished products—e.g., combs, fittings, spikes or gaming pieces. The antlers could have been acquired from hunted animals or from seasonal drops found on the ground [40,41]. Due to the fact that deer remains are very rarely found, it can be assumed that such material was a rarity in workshops, and its low availability might have affected the final price.

## 5. Conclusions

Based on archaeozoological data, wild animals did not play any significant role in the economy of medieval and early modern Wrocław, but their use remained at a low yet stable level.

The frequency of wild animal bones in the archaeozoological material indirectly determines the condition of the biotope around Wrocław, confirming the previous opinions about its field-and-woodland character. The attendance of easily adapting species such as boars, hares and roe deer indicates the existence of fields. The remains of red deer and wisent suggest the presence of woodlands. In the early modern era, we can observe a slight decrease in the amount of wild game, suggesting intensified development of settlements around Wrocław and growth in the farming economy.

A relatively higher share of wild animals in the materials from medieval Ostrów Tumski is a sociotopographic indicator and confirms the elite character of the settlement, which was a seat of clerical and secular power. Based on the anatomical analysis of the remains of wild animals from św. Idziego Street (Cathedral Island), certain butchering practices and consumption preferences of the inhabitants of Ostrów Tumski were observed. Probably all parts of the hare carcass, parts closer to the pectoral limb and parts of the boar’s head were preferred. A higher number of deer head bones may indicate the acquisition of antlers; however, these conclusions are ambiguous due to the low number of bones tested.

The left-bank settlement was inhabited by craftsmen and merchants of various social statuses who did not have hunting rights. Evidence of the presence of wild game in this part of Wrocław in medieval times may indicate breaching of hunting laws/regalia. The meat of wild animals has appeared at market stalls since the 15th c., as confirmed by the written remarks about venison vendors. On the basis of the tax book, venison vendors did not fulfil a very substantial economic and social function; however, the scarcity of sources prevents us from making any broader conclusions.

Items manufactured from bones, antlers and hides of wild animals were relatively rarely produced and used by the city inhabitants in contrast to items made from the materials acquired from domesticated animals. The use of such material probably raised the price and, consequently, gave the product a more luxurious character.

## Figures and Tables

**Table 1:** Chronology and location of individual archaeozoological materials from archaeological research in Wrocław.

Location	Chronology	Literature
Nowy Targ Square	11th–15th century	Chrószcz, Janeczek, Pasicka 2018 [1]
4 Katedralna Street, Ostrów Tumski (Cathedral Island)	11th–12th century	Chrószcz, Janeczek 2012 [29]
4 Katedralna Street, Ostrów Tumski (Cathedral Island)	15th–17th century	Chrószcz, Janeczek 2012 [29]
4–6 Św. Idziego Street, Ostrów Tumski (Cathedral Island)	11th century	Chrószcz, Janeczek, Paradowski, Sudoł 2015 [30]
4–6 Św. Idziego Street, Ostrów Tumski (Cathedral Island)	12th to 1st half of the 13th century	Chrószcz, Janeczek, Poradowski, Sudoł 2015 [30]
50 Rynek (Market Square)—18 Igielna Street	2nd half of the 13th century	Wiszniowska, Stefaniak, Socha 2002 [32]
Rynek (Market Square)	2nd half of the 13th to 1st half of the 14th century	Wiszniowska, Stefaniak, Socha 2001 [31]

**Table 3:** Chronology and location of archaeological materials from excavations in Wrocław.

Location	Chronology	Stuff	Literature
Nowy Targ Square	12th–18th century	Leather and antlers	Radek 2018 [38] Gomułka 2018 [39]
Katedralna Street, Ostrów Tumski (Cathedral Island)	11th–12th century 15th–18th century	Antlers	Jaworski 2012 [40]
Św. Idziego Street, Ostrów Tumski (Cathedral Island)	11th–13th century	Leather, bones and antlers	Konczewska, Radek 2015 [37] Jaworski 2015 [41]
Szewska Street	2nd half of the 13th to the 14th century	Leather	Konczewska 2010 [46]

**Table 4:** Percentage of wild animals from Ostrów Tumski (Cathedral Island) with distinction between *animalia magna* and *animalia minuta* over the centuries [29,30] (Description: M. Pietruszka).

	Species	Chronology				
		11th c.	11th–12th c.	12th–13th c.	12th to 1st half of the 13th c.	15–17th c.
Animalia magna	*Capreolus*	10.30%	5.30%	14.75%	14.80%	32.80%
	*Cervus*	12.60%	67.30%	4.92%	4.90%	10.80%
	*Bison*	6.90%	7.90%		9.80%	5.60%
	*Ursidae*	3.40%		4.92%	4.90%	
	*Sus scrofa*	9.20%		55.47%	55.70%	2.80%
Animalia minuta	*Sciurus*	5.70%	2.60%			1.60%
	*Leporidae*	47.10%	13.70%	6.56%	6.60%	39.60%
	*Vulpes*	3.40%		3.28%	3.30%	2.80%
	*Castor*	1.10%	2.60%			
	*Dama dama*					1.20%

c.: century.

**Table 5:** Anatomical distribution of wild animals at the site św. Idziego Street (Cathedral Island) 11th–13th c. [29]. Description: M. Pietruszka.

	*Bison*	*Capreolus*	*Cervus*	*Lepus*	*Sus Scrofa*	*Vulpes*	*Ursus*	*Castor*
Cranium				1	3	1	1	
Processus cornualis			5					
Mandibula		4	2	1	12	2		
Dentes					21	1		
Vertebrae		1	1	6				
Costae/Os coracoideum				2				
Scapula	1	5		3			1	
Humerus	1		1	9	2	1		
Radius	1		1	3				
Radius et Ulna	1		1					
Ulna				1				
Phalanxproximalis	5	7	1		1			
Phalanxdistalis							1	
Pelvix/Synsacrum				12	2		1	
Femur				5				1
Tibia/Tibiotalus		1		6				
Fibula/Os maleolare			1					
Talus								
Os tarsi/Tarsometatarseus	1				1		1	
Os tarsicentroquartale	1							
Os metatarsale	1							

c.: century.

## Data Availability

The collected data were collected and brought together from individual studies published in the literature given in the references.
